# SOCIAL ISOLATION, LONELINESS, AND MATTERING AMONG OLDER ADULT FACEBOOK USERS FROM DIVERSE BACKGROUNDS

**DOI:** 10.1093/geroni/igac059.122

**Published:** 2022-12-20

**Authors:** Jess Francis, Simon Brauer

**Affiliations:** University of Michigan, Ann Arbor, Michigan, United States; University of Michigan, Ann Arbor, Michigan, United States

## Abstract

Older adults who are childless and live alone are at risk for social isolation and loneliness. Studies show Facebook communication to be associated with feelings of mattering – the feeling that we are significant to others - a protective psychosocial resource. Despite being at similar stages in life, older adults, their technology use, and their experience of social isolation can vary greatly. This study explores how older adults from different demographic and socioeconomic backgrounds use Facebook and how that use is associated with perceptions of mattering, loneliness, and social isolation. This study employed online survey methodology among a sample of older adult Facebook users (n = 517; Mage=70; 50% Female; 92% White). Preliminary results show significant differences among income level, age, and gender regarding Facebook communication and mattering among older adults who are childless and live alone. We discuss our full results in tandem with qualitative data from our sample.

